# Successful Management of Triplet Heterotopic Pregnancy (Interstitial) With an Intrauterine Monochorionic Diamniotic Twin Pregnancy Through Laparoscopy: A Case Report

**DOI:** 10.7759/cureus.37249

**Published:** 2023-04-07

**Authors:** Nihar R Bhoi, Vipin Chandra, Kshitiz Murdia, Kishor Kawad

**Affiliations:** 1 Reproductive Medicine, Indira In Vitro Fertilization (IVF) Hospital Private Limited, Udaipur, IND; 2 Clinical Research and Operations, Indira In Vitro Fertilization (IVF) Hospital Private Limited, Udaipur, IND; 3 Reproductive Medicine, Indira In Vitro Fertilization (IVF) Hospital Private Limited, Ahmedabad, IND

**Keywords:** ivf/icsi, dual embryo transfer, laparoscopy, interstitial, monochorionic diamniotic pregnancy, heterotopic pregnancy

## Abstract

The simultaneous occurrence of intrauterine (IU) and extrauterine pregnancies is known as heterotopic pregnancy, an uncommon clinical condition that is challenging to manage. It can be a potentially fatal illness if it remains unnoticed. This is a case report of a woman who had heterotopic triplets after transferring two embryos produced through in vitro fertilization. An ultrasound scan diagnosed live interstitial heterotopic pregnancy and an intrauterine monochorionic twin pregnancy. Laparoscopic resection of interstitial heterotopic pregnancy was done. The monochorionic twin pregnancy was closely monitored by serial ultrasound, and at 36 weeks of gestation, two healthy twins were delivered by cesarean section. The fetal growth parameters were monitored, and a dopplers study was conducted to assess fetal blood flow. Even in heterotopic pregnancy, timely diagnosis and therapeutic intervention can preserve IU pregnancy with a successful outcome. Early meticulous monitoring and early detection can lead to a favorable outcome. Even in heterotropic pregnancy, a meticulous evaluation can lead to favorable outcomes by conserving IU pregnancy, and timely intervention can prevent maternal motility.

## Introduction

Heterotopic pregnancy is rare and is mostly seen with more than one gestation: one inside the uterus and one outside it, most frequently in the fallopian tube but less frequently in the cervix or ovary. The incidence of heterotopic pregnancy is around 1/30,000 (1/10000 to 1/50000); however, the incidence is higher with assisted reproductive procedures, ranging from 1/100 to 1/3600, and has been as high as 1% in some studies [1]. There are few reports of interstitial ectopic and concurrent monochorionic twin intrauterine (IU) pregnancy [2,3]. Heterotopic pregnancy is life-threatening to the mother’s life and may be dangerous for IU pregnancy [4]. This case report illustrates a heterotopic triplet with interstitial ectopic and IU monochorionic twin pregnancy. The vital aspect of this case report was the timely diagnosis and laparoscopic management of heterotopic pregnancy as early as 6-7 weeks of gestation [5].

## Case presentation

A 35-year-old nulligravida with an active married life of six years came for infertility treatment. On initial screening, all reports of the husband were found normal, and blood reports of females were normal. The ultrasound scan showed diminished ovarian reserve, bilateral endometrioma, and subserous fibroid of 6x7 cm. So, the couple was counseled for laparoscopic removal of bilateral endometrioma and myomectomy, followed by in vitro fertilization (IVF)/intracytoplasmic sperm injection (ICSI). On doing laparoscopy, both tubes were adherent to the pod and respective ovaries and showed terminal hydrosalpinx. So, clipping of both the fallopian tube was done. Downregulation was done with two injections of Goserelin acetate 3.6 mg depot to overcome the residual effect of endometrioma. Endometrial preparation was done with estradiol valerate 2 mg once a day. Two grade 1 (4AA, 5AB) blastocysts were transferred under ultrasound guidance. β-hCG report was done after two weeks which was positive with a value of 2147 mIU/ml.

**Figure 1:**
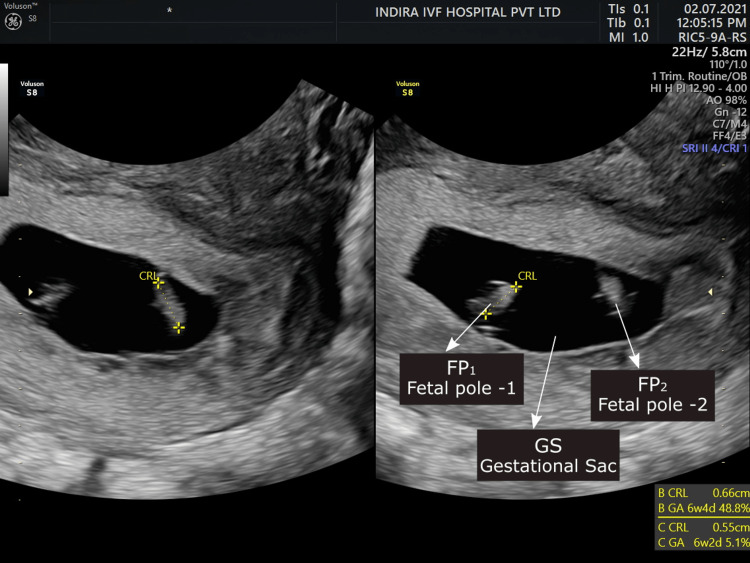
Ultrasound scan showing intrauterine live monochorionic twin pregnancy. FP1: Fetal pole 1; FP2: Fetal pole 2; GS: Gestational sac.

**Figure 2:**
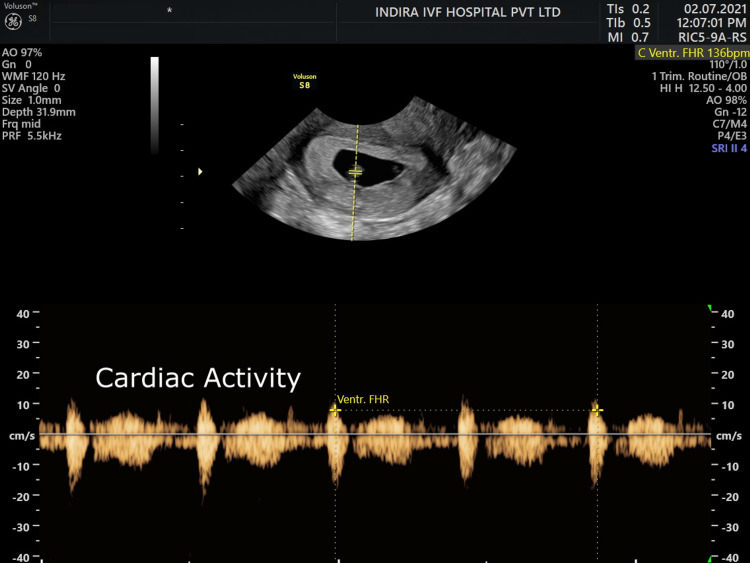
Ultrasound scan with fetal pole and cardiac activity.

After two weeks of the β-hCG report, an ultrasound scan was done, which showed IU live monochorionic twin pregnancy (Figures 1-2).

Another gestational sac with fetal pole and cardiac activity was seen at the left adnexa near the cornual end showing triplet heterotopic pregnancy (Figure 2). As it was live ectopic pregnancy in the left cornual end, the decision of laparoscopic removal of that pregnancy was taken in view of the prognosis of IU pregnancy.

**Figure 3:**
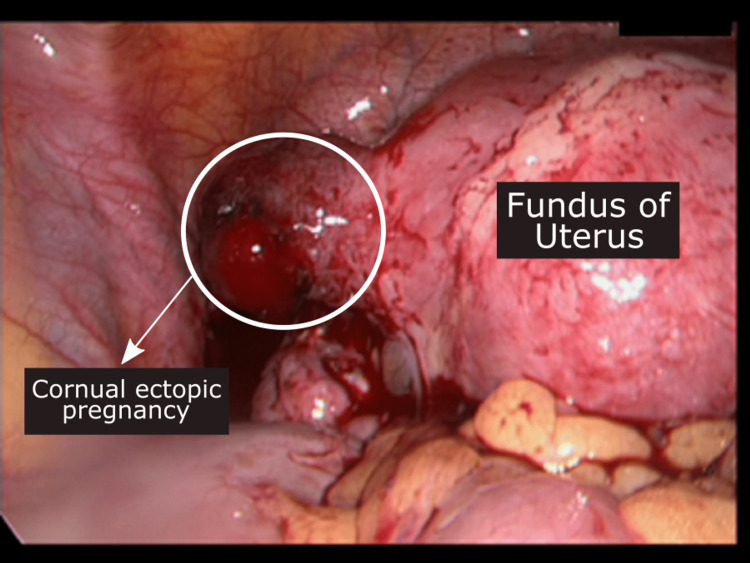
Laparoscopic removal of left cornual ectopic pregnancy.

The patient was taken for surgery under general anesthesia (GA) using propofol. She was placed in a modified lithotomy position. Primary trocar (10 mm) was done by direct trocar entry technique through the supraumbilical site (2 cm above the umbilicus), and two accessory ports were created under vision on the contralateral side. The intraabdominal pressure was maintained at 12 mmHg pressure. The intraoperative finding (Figure 3) was in accordance with the ultrasound finding suggestive of cornual pregnancy. The mass (ectopic) was approximately 2.5-3 cm in diameter and was angry looking. A decision for cornual resection was taken. The bleeding points were secured by bipolar electro-coagulation. The current setting was kept low at 25 watts to minimize the effect on the IU pregnancy. The resected specimen was collected in an endo-bag, and retrieval was done by widening the 5 mm port. The specimen was sent for histopathological examination. On the next day of surgery, the viability of the IU twin pregnancy was confirmed by ultrasound. Isoxuprine, 40 mg in 500 mL dextrose solution, was given at a rate of 80 µgm/min for 24 hours. The dose was titrated, keeping the pulse rate from 110 to 120 bpm. Intramuscular progesterone 100 mg IM once daily for three days was given before switching to vaginal gel (8% once daily).

Monochorionic diamniotic pregnancy was confirmed on nine weeks ultrasound scan. The nuchal translucency scan (at 12 weeks of GA), anomaly scan (19 weeks of GA), and fetal echo (at 22 weeks of GA) of the IU twin pregnancy were normal. Pregnancy was monitored closely to diagnose any complications of monochorionic twin pregnancy. Growth scans with doppler studies were normal at 32 and 34 weeks of pregnancy. After 36 weeks, the pregnancy was terminated with a lower segment Caesarean section (LSCS). Two healthy male children of 2.2 kg and 2 kg were delivered without complications. The neonatal period was uneventful except for physiological jaundice, which was cured with phototherapy only.

## Discussion

Heterotopic triplet pregnancy is a very rare medical condition seen in day-to-day practice. However, because of the increased use of assisted reproductive technology (ART) procedures, the incidence of ectopic and heterotopic pregnancy has increased [4]. The factors that are responsible for the occurrence of heterotopic pregnancy are the same as those of ectopic. The attributable causes are multiple embryo transfers, transfers around the uterine horn, high syringe pressure, deep catheter penetration during transfer, and IU adhesions. The most common presenting features are amenorrhea, spotting, pain abdomen, and fainting attack. The symptoms may differ according to ruptured or unruptured pregnancy [5]. However, ectopic pregnancy may be seen as a non-specific adnexal mass with or without free fluid in a pouch of Douglas (POD). The doppler study might show increased vascularity around the sac, mimicking a ring of fire. Transvaginal sonography (TVS) has recently made strides that have aided in the early detection of heterotopic pregnancies [6]. The sensitivity of the TVS to identify heterotopic pregnancy at 5-6 weeks is only about 56% [6]. An IU pregnancy and an ectopic gestational sac with a fetus are typically seen in TVS of the uterus when heterotopic pregnancy is present [7]. In our case, there was an IU twin monochorionic pregnancy with coexisting gestational sac with fetal pole and cardiac activity seen in the left adnexa near the cornual end. The patient had no signs and symptoms of heterotopic pregnancy. Heterotopic pregnancy was diagnosed on transvaginal ultrasound scan 15 days after β-hCG positive report. The interstitial ectopic pregnancy was surgically removed with limited manipulation of the uterus. IU pregnancy was monitored and closely followed up till 36 weeks. The outcome was the birth of two healthy babies. The vital aspect of this case report was the timely diagnosis and laparoscopic management of heterotopic pregnancy as early as 6-7 weeks of gestation. It is also advisable that in IVF treatment, the number of embryo transfers should be limited to a maximum of two embryos.

Due to the rarity of heterotopic pregnancy, precise, timely diagnosis is typically hard. A delay or non-observance could result in potentially life-threatening complications such as ruptured ectopic pregnancy, hypovolemic shock, or even death; therefore, an early and accurate diagnosis of heterotopic pregnancy is vitally crucial [8]. The TVS examination is highly sensitive in the conclusive diagnosis of heterotopic pregnancy. A routine TVS 4-6 weeks following ART is suggested to exclude ectopic and heterotopic pregnancy [9]. The treatment of heterotopic pregnancy tries to save the IU pregnancy and remove the ectopic interstitial pregnancy using different therapeutic techniques: surgical, medical, and expectant techniques [10]. Expectant management could be used for patients with a stable hemodynamic state and who are asymptomatic; however, strict periodic monitoring is mandatory. It avoids potential risks associated with surgery and transabdominal sonographic-guided aspiration of the ectopic gestational embryo [11,12]. Surgical management includes either laparotomy or laparoscopy as viable therapy options for heterotopic pregnancy [13]. Emergency surgery is strongly advised for individuals with an unstable hemodynamic state or any symptoms of ectopic pregnancy. Different surgical procedures such as salpingectomy, oophorectomy, salpingostomy, cornual resection, and even total abdominal hysterectomy are used to remove an ectopic pregnancy mass [14,15]. Some embryo-toxic medicines, such as potassium chloride and hyperosmolar glucose, are used for embryo-killing [14,16]. In conservative therapy of ectopic pregnancy, MTX is widely used; however, MTX-related teratogenicity in surviving IU fetuses following failed medical abortion or alternative treatment has already been found [16]. MTX should be avoided while dealing with heterotopic pregnancy [16]. Therefore, regular ultrasound examinations and thorough monitoring are necessary. It is recommended for early evaluation and monitoring to ensure a good maternal outcome.

## Conclusions

In conclusion, as the rate of ectopic and heterotopic pregnancy is increasing, such essential measures should be taken to avoid fatal effects. So, any patient with ectopic/heterotopic pregnancy or who has undergone ovulation induction or any type of ART should be suggested a transvaginal ultrasound scan and laparoscopic management, which can save patients' lives and coexisting IU pregnancy.
